# Detecting Lombard Speech Using Deep Learning Approach

**DOI:** 10.3390/s23010315

**Published:** 2022-12-28

**Authors:** Krzysztof Kąkol, Gražina Korvel, Gintautas Tamulevičius, Bożena Kostek

**Affiliations:** 1 PGS Software, 50-086 Wrocław, Poland; 2 Institute of Data Science and Digital Technologies, Vilnius University, LT-08412 Vilnius, Lithuania; 3 Audio Acoustics Laboratory, Faculty of Electronics, Telecommunications and Informatics, Gdańsk University of Technology, Narutowicza 11/12, 80-233 Gdańsk, Poland

**Keywords:** Lombard speech, 2D feature representations, threshold-based averaging strategy, speech recognition, machine learning

## Abstract

Robust Lombard speech-in-noise detecting is challenging. This study proposes a strategy to detect Lombard speech using a machine learning approach for applications such as public address systems that work in near real time. The paper starts with the background concerning the Lombard effect. Then, assumptions of the work performed for Lombard speech detection are outlined. The framework proposed combines convolutional neural networks (CNNs) and various two-dimensional (2D) speech signal representations. To reduce the computational cost and not resign from the 2D representation-based approach, a strategy for threshold-based averaging of the Lombard effect detection results is introduced. The pseudocode of the averaging process is also included. A series of experiments are performed to determine the most effective network structure and the 2D speech signal representation. Investigations are carried out on German and Polish recordings containing Lombard speech. All 2D signal speech representations are tested with and without augmentation. Augmentation means using the alpha channel to store additional data: gender of the speaker, F0 frequency, and first two MFCCs. The experimental results show that Lombard and neutral speech recordings can clearly be discerned, which is done with high detection accuracy. It is also demonstrated that the proposed speech detection process is capable of working in near real-time. These are the key contributions of this work.

## 1. Introduction

The Lombard effect occurs when a human speaker unconsciously increases their vocal efforts to retain the level of speech intelligibility in noisy conditions [1]. These vocal efforts alter speech production and may affect the pitch of the speech signal, phoneme duration, spectral tilt, and the overall energy level, including formant frequency shift [2]. In addition, the Lombard effect is person-, gender-, age-, as well as noise-level dependent [2,3]. Moreover, the auditory outcome of the Lombard effect may be similar to emotional speech, which is characterized by variability in pitch, phoneme durations, energy level, spectral tilt, etc. Thus, the resulting characteristics of Lombard speech are ambiguous and difficult to formalize and build a mathematical model upon.

Detecting Lombard speech in adverse speech-in-noise conditions is a challenging task. Even more demanding is to create an algorithm designated for public address systems, broadcasting systems, emergency voice communication systems, hearing aids, etc., that operates in near or real time [4,5]. It is evident that the more complex the algorithm, the more time it takes to perform the analysis [6], resulting in delays. Due to the complex nature of the Lombard effect [7], deep learning techniques seem more suitable than baseline algorithms, being successfully applied in real-time speech recognition systems [8,9]. Therefore, in our work, we propose deep neural network models that are relatively simple. Even though, no matter how simple a model we choose, performing an analysis on a large set of acoustic features converted to two-dimensional (2D) space in real time requires high computational costs. Therefore, a threshold-based strategy for averaging the Lombard effect detection results is introduced to reduce the computational cost and not resign from the 2D representation. It is also assumed that training the neural network may be long as it does not affect the speed of recognizing the type of speech signal. An additional requirement is the availability of sufficiently short but Lombard-based, prosody-varied speech signals for training. Thus, it is decided that continuous speech, not isolated patterns of segmented speech (such as one word), is to be used for neural network training. Overall, we believe that the proposed threshold-based averaging strategy expands research dealing with the Lombard effect detection.

In the presented work, the following are the key contributions:

A data augmentation technique determining characteristics related to the speaker’s gender is proposed.

The effectiveness of different CNN model structures with various feature representations is explored in the context of detecting Lombard speech.

A threshold-based strategy for averaging the Lombard effect detection results is proposed and evaluated in near real-time conditions on German and Polish recordings.

It should also be emphasized that the platform on which processing and detection are performed significantly impacts inference speed [6]. Therefore, for this work, it was assumed that the detection process of a single recording performed on a personal computer should take no more than the recording itself.

The Lombard effect is known to impact the performance of speech recognition systems unfavorably. Various researchers have analyzed Lombard speech produced in different types and levels of noise for speech intelligibility [10,11,12], audio and audio-visual speech recognition [13,14,15,16], speaker recognition [17,18,19], and emotional speech analysis [20]. Overall, an automatic speech recognition system (ASR) performance may be degraded when Lombard speech is present in the speech signal [15,16,21,22,23,24]. This refers to the case when the ASR system is not trained on Lombard samples but only on “normal” (neutral) utterances. Moreover, speech recordings are conditioned upon the space constraints in which they are registered or collected [25]. In contrast, in the case of human-to-human communication, the Lombard effect improves speech intelligibility in noise [26,27,28], while for human-to-computer communication, it increases the already high variability of the speech signal. Pursuing these two seemingly contradictory goals is, therefore, complex and complicated to solve.

Generally, the Lombard speech detection process may be implemented using different approaches. The simplest one concerns a typical signal-based method, i.e., the fundamental frequency (F0) value can be calculated and verified if it is above some—empirically assigned/calculated—threshold level. Apparently, the increased F0 value is one of the most significant features of Lombard speech. This method, however, has some limitations, i.e., without any reference, the level of F0 that can be considered as having increased cannot easily be defined. Moreover, the fundamental frequency differs for male and female voices; therefore, it might be impossible to define any decision level without determining the speaker’s gender first. Furthermore, as already mentioned, the speaker’s emotional state may be another F0 variation source. High-arousal emotions such as anger, excitement, and joy cause a rise in the average F0 value, which can be misunderstood as the Lombard effect when automatically analyzing speech signals. Considering the above, Lombard speech detection requires a machine-learning approach, specifically deep models, as they are currently use in state-of-the-art automatic speech recognition (ASR) [29] and human–computer interaction (HCI) systems [30,31]. Trabelsi et al. indicated that even though ASR-designated high-end technology is available, e.g., Google Assistant or Amazon’s Alexa, it cannot deal with new accents, vocabularies, and customized solutions [29]. They suggest building personalized models based on well-known open-source tools such as Deep Speech [32] or Kaldi [33], or new datasets can be employed [34]. It should, however, be noted that many ASR papers are related to speech-to-text solutions or voice-to-voice converters [35,36,37]. In contrast, Lombard speech detection does belong to the category of speech enhancement methods [38,39,40].

In a recent paper by Nossier et al. [38], it was pointed out that investigating the Lombard effect in the context of preprocessing techniques needs further attention and research as this may be one of the effective speech enhancement methods [38]. This is one of the critical studies as it shows the advantage of employing deep models for removing noise that is accompanied by the target speech signal, even though it requires much more data compared with baseline algorithms [38].

Given the complexity of the acoustic manifestation of the Lombard effect, it is impractical to analyze only selected speech signal parameters. It is necessary to explore all the signal properties affected by the Lombard effect [39]. Hence, temporal, spectral, cepstral, and chroma-based analysis techniques can be used for this purpose [41,42,43]. Feature vectors may be derived from the speech signal as a whole or divided into smaller units, e.g., allophones [44,45]. Speech signal analysis may also result in the form of a two-dimensional (2D) space feature [46], as such a format is suitable for a machine-learning-based approach, and it usually outperforms the one-dimensional representation [47]. By applying machine learning algorithms, we can expect automatic identification of trends and regularities of the extracted features, nonlinear modeling capabilities (which is the case for speech signals), adaptation, and improvement capabilities. In this case, the detection of the Lombard effect can be treated as a classification task. Many researchers have performed different classification tasks on various speech phenomenon detection, such as speech emotions [48,49] and Parkinson’s and other diseases [50,51,52]. Because 2D deep learning techniques have been repeatedly shown to achieve promising results, therefore, in this work, we used a two-dimensional convolutional neural network (CNN) to build a deep neural network (DNN) classifier. In contrast to other research combining CNNs with 2D representations of speech, we extend the standard implementation by using a specific augmentation. Since it is known that the Lombard effect affects speech/speaker recognition performance [27], it is therefore expected that adding speaker information for augmentation purposes would help to improve the performance of the process.

The remainder of the paper is organized as follows. It starts with a description of the experimental framework used for Lombard speech detection and its stages, i.e., generating 2D speech representation, proposing a deep model structure, and introducing the Lombard effect detection averaging method. Section 3 describes the experiments performed and their results. The focus of the result analysis is on the evaluation of the Lombard speech detection process effectiveness and a discussion comparing the experiment outcome with state-of-the-art achievements. This is followed by the conclusion section (Section 4) summarizing the key points of this study, its limitations, and the future direction of Lombard speech detection development.

## 2. Lombard Speech Detection Process

Speech type detection may be treated as a binary classification problem—in other words, speech can be “Lombard” or “non-Lombard” (i.e., “normal”). In reality, the speech signal is way more complicated than just simple Lombard vs. non-Lombard differentiation. One may even argue about the definition of “normal” speech [22]. For example, silence or unvoiced fragments may occur, or there may be a mixture of speech, etc. Moreover, rarely is it known whether recordings are collected in a controlled environment [22]. Therefore, the detection is always an approximation of the speech type; however, incorporating an averaging procedure allows for building a near real time Lombard speech detection process.

**Figure 1:**
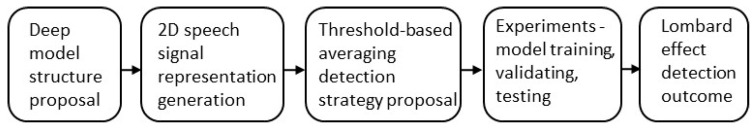
Flowchart of the Lombard speech detection process.

This section presents the proposed Lombard speech detection process consisting of a detection model and a threshold-based strategy for averaging the short-time Lombard speech detection results. The flowchart of the experimental framework performed is presented in Figure 1.

### 2.1. Deep Model Structure

The deep model proposed for the detection of Lombard speech is convolutional neural networks (CNNs). CNN is a regularized multilayer perceptron that takes advantage of the hierarchical data, applying convolutional filters to determine which parameters are to be defined in the process of learning [53]. The 2D CNN is a type of neural network designed to maintain the spatial integrity of the processed image. CNN treats 2D representations differently; namely, it extracts features from the processed image by sliding a convolutional filter over an image and calculating the feature maps. These feature maps create a set of new pixel values calculated from the source image and filter. Convolutional filters applied to the source image are characterized by the following attributes:

- Size of the filter tensor (it is usually two- or three-dimensional since the image might be greyscale or color);
- Stride—the number of pixels by which the filter is moved in the subsequent steps;
- Padding—whether the resulting pixel set should be padded with empty pixels to retain the exact size of the feature map as the source image;
- The number of filters that should be applied to the image.

**Figure 2:**
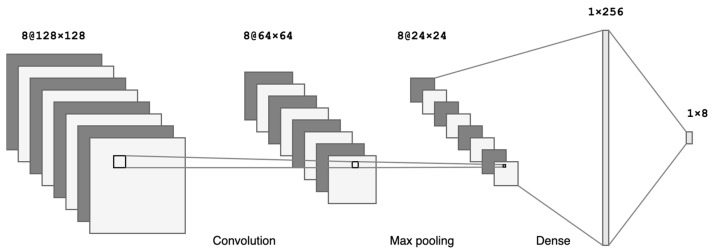
CNN network with three convolutional layers with max pooling and one dense layer and classification output.

A block diagram of the convolutional neural network is presented in Figure 2.

Each unit in the structure of CNN receives input from other units in its neighborhood. This means that the network focuses on local data changes and allows for simple detection of edges, contrasting areas, and similar features in speech visual representations [54].

### 2.2. 2D Speech Signal Representation

The detection process involves recognizing the speech type using a typical classification model with two output classes: Lombard and non-Lombard. Various 2D feature representations of speech signals are verified to identify the best combination of CNN and 2D speech representation. The overall idea is as follows: since the speech signals should be analyzed in near real-time conditions, the feature represented in the form of the image might help detect the character of the given fragment of speech. A neural network that detects the type of speech based on the relevant speech representation with high accuracy may be applied in a decision pipeline that determines whether to modify speech further or not, because it already has the Lombard effect-like features.

**Figure 3:**
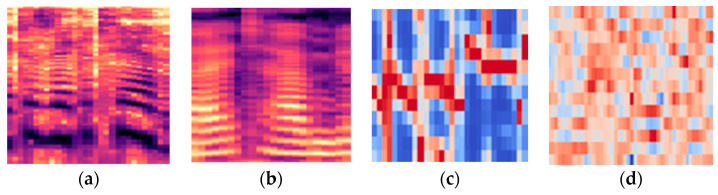
The 2D feature representations of speech signal: (**a**) spectrogram, (**b**) mel spectrogram, (**c**) chromagram, (**d**) MFCC-gram.

The time-frequency signal features are converted to the following 2D representations: spectrogram, mel spectrogram, chromagram, and MFCC-gram. Visualization of several types of 2D feature representations is shown in Figure 3, and they are shortly described in this subsection.

(1) Spectrogram

A spectrogram is a visual 2D representation of the signal energy distribution in frequency and time domains. Let:

(1) $$x\;=\;{\left[x\left(1\right),x\left(2\right),\ldots ,x\left(N\right)\right]}^{T}$$

be a sequence of samples of the analyzed speech signal, where N is the number of samples per signal and the T superscript placed on the matrix (i.e., ${\left[.\right]}^{T}$) refers to the matrix transpose operation.

The spectrogram construction process is based on calculating the short-time Fourier transform (STFT) for this speech signal. The magnitude spectrum of the l-th short-time segment (denoted by $X_{l}$) is obtained by the following formula:

(2) $$\left|X_{l}\left(k\right)\right|\;=\;\frac{1}{M_{FT}}\sqrt{{\left(X_{l}\left(k\right)\right)}_{re}^{2}\;+\;{\left(X_{l}\left(k\right)\right)}_{im}^{2}}$$

where $X_{l}\left(k\right)$ is the Fourier transform of the short-time segment $x_{l}$, $k\;=\;1,\;\ldots ,M_{FT}$ ($M_{FT}$ refers to the number of Fourier transform coefficients) and l = 1, …,L (L refers to the number of short-time segments).

(2) Mel spectrogram

Mel spectrogram is a mel-scaled power spectrogram. For this purpose, the mel filter bank is constructed over the frequency range from the lower to the upper frequency. The mel spectrum is obtained by multiplying the spectrum coefficients by the filter coefficients. The relationship between the mel scale and the Hertz scale can be described by the following formula:

(3) $$Mel\left(f\right)\;=\;2595\log\left(1\;+\;\frac{f}{700}\right)$$

where f is a given frequency in Hertz. The mel scale is fundamental in applications of speech processing because it reflects our perception of sound.

(3) Chromagram

A chromagram is another type of representation (and visualization) where the entire spectrum is projected onto 12 bins representing the 12 semitones of the musical octave. As discussed by Müller [55], the human perception of the pitch has a “color” periodicity, which means that two pitches are perceived as similar (in their “harmonic role”) if they differ by an octave. This resulted in an observation that every pitch might be represented by two factors: tone height and chroma. Tone height is represented by the octave number, while the chroma is the number of pitches inside the octave (0 to 11), just like sounds in a chromatic scale (C–C#–D–D#–…–B).

A chromagram can be created by summing up all coefficients belonging to the same chroma, and it is derived from a pitch-based log-frequency spectrogram having 127 coefficients. Due to its “musical” context, it does not fit well with the speech visualization problem.

(4) MFCC-gram

Mel-frequency Cepstral Coefficients (MFCCs) are a compressible representation of the mel spectrogram. To obtain MFCCs, a log magnitude of the mel spectrum is calculated, and then discrete cosine transformation (DCT) is applied. The mathematical expression of MFCCs is as follows:

(4) $$c_{n}\;=\;\overset{M\;-\;1}{\underset{i\;=\;0}{\sum }}m_{i}\cos\left(\frac{\pi n(i\;+\;1/2)}{M}\right)$$

where $m_{i}$ are the log filter bank amplitudes, and M is the number of filters, n = 1, …,M − 1.

### 2.3. Threshold-Based Strategy of Averaging the Lombard Effect Detection Result

It should be noted that detecting and recognizing the type of speech should be dynamic as one speaks and does not involve a longer fragment of speech but only a short piece of it (e.g., 0.25 s). Often, it is a fragment that does not carry too much energy (e.g., a moment of silence), or there may be an ambiguous fragment (e.g., one in which a large part of the time contains silence and non-energetic phonemes), so it is a high probability that the nature of such speech changes as the sentence is uttered. Therefore, it should be taken into account that the detection/recognition of the speech type would not be the same for the entire course of the tested speech signal. To avoid misclassification due to a temporary change in the character of the utterance, averaging the results is a critical element of the recognition process; hence, in our work, it is applied to the entire recording, however, it is based on an assigned threshold. In the case of real-time detection and recognition, the process memory can be used, e.g., averaging the results for the last dozen or so windows.

**Figure 4:**
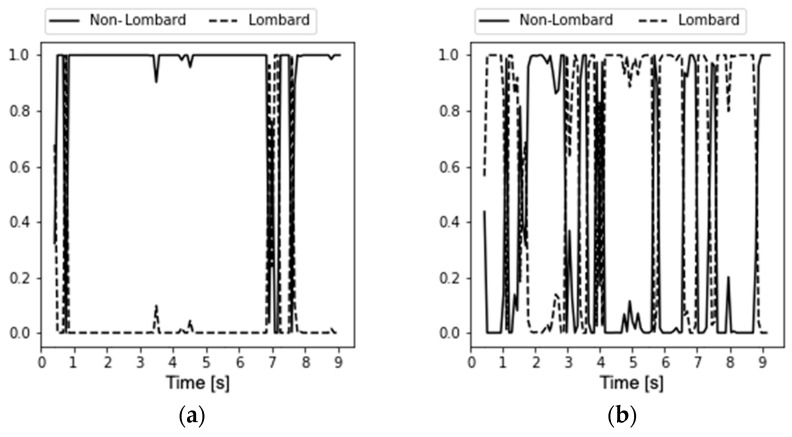
Sample detection results for (**a**) neutral speech recording and (**b**) Lombard speech recording.

A graphical representation of the importance of averaging the results is given in Figure 4, where an example of sample detection results is presented. On the horizontal axis, there is the number of the classified frame, and on the vertical axis, the probability of the fact that a given window is a fragment of Lombard (dashed line) or neutral (solid line) speech is shown.

The left side of Figure 4 shows a typical recording of non-Lombard speech and most of the frames of the recording have been classified as neutral. The right side of Figure 4 shows a recording of the Lombard speech. The fluctuations are much greater, but the advantage of frames classified as Lombard speech is visible. From the point of view of the classification of the recording as a whole, average detection is essential. A procedure for averaging the Lombard effect detection results is performed by the following pseudocode, shown in algorithmic form (Algorithm 1):

**Algorithm 1:** A procedure for averaging the Lombard effect detection results.INPUT: vector $\left[N,\;L\right]$ **If**
N > L
**then** the frame is non-Lombard **else**
the frame is Lombard-like **end if** OUTPUT: the value of NOISE

The comment on the algorithm:

- It was assumed that the threshold for classifying a given frame as Lombard is 0.5, i.e., if the neural network returns the vector $\left[N,\;L\right]$ for each frame, it returns two probabilities: probability N, when the given frame is neutral speech; probability L, in the case of Lombard speech.
- For NOISE = 0, neutral speech, for NOISE = 1, Lombard speech.

The results of the above algorithm are collected into the vector X, the length of which depends on the number of classified frames. It should be mentioned that all frames are classified whether or not they contain speech. The classification of the entire recording will result in the average of $A\;=\;AVG\;\left(X\right)$. The obtained value is then compared with an empirically defined threshold level Y. This level is called the cutoff level, and the classification result is determined according to it.

## 3. Experiments and Result Analysis

To test the effectiveness of our proposed method, we performed its validation on two datasets: German [56] and Polish [57]. In this section, we introduce the process of preparing these recordings and then discuss the experimental implementation. The main goal of the experiment is two-fold: first, to check the effectiveness of different CNN model structures with various feature representations and then to implement the Lombard speech detection process and evaluate its performance. Therefore, the experimental layout and results are reported in two subsections.

### 3.1. Experimental Setup

**Figure 5:**
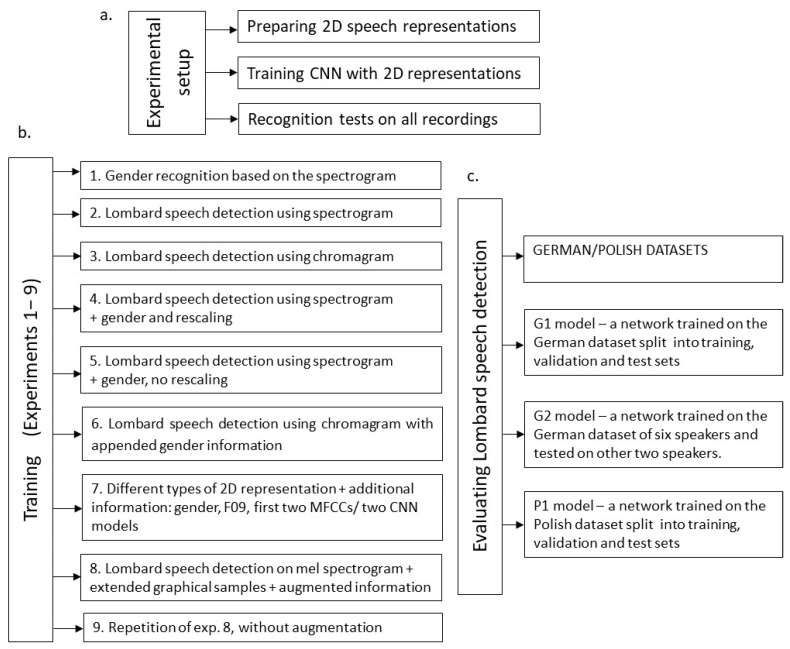
Experimental setup: (**a**) overall experiment design, (**b**) various configurations of 2D feature space representation augmented by additional information combined with CNN models and datasets employed in training, (**c**) evaluation stages.

The experimental setup overview is shown in Figure 5a. Various configurations of 2D feature space representation augmented by additional information, such as the speaker’s gender, are combined with CNN models and datasets employed in training (see Figure 5b) and evaluation stages (Figure 5c).

### 3.2. Preparation of Recordings

For the purpose of training and inference, two sets of recordings in two languages were used. Information about the sets implemented is given in Table 1.

All sets contain recordings of neutral and Lombard speech, which made it possible to segment the recordings, label them, and use them in the network training in the supervised learning process. The process of preparing recordings includes the following steps:

***Step 1.*** Calculating STFT and amplitude value, and the window length is 512 samples. The hop length is set to half of the window length.

***Step 2.*** The next step is to truncate the first 10 spectrum values. Practically, they do not carry any information, and they add a lot of noise to the spectrogram.

***Step 3.*** The next step is to remove all voiceless fragments from the spectrum, i.e., those where there is essentially no energy. The effect is that if less than 90% of the content of a given window does not carry information about the speech signal (it is voiceless or simply silent or disturbed), then such a window is not included in the training. The point is that the silence window can misclassify a given type of speech.

***Step 4.*** The last step is to generate the visualization and save it as an image (png), scaled to such a resolution that it is effective for the training algorithm (too high resolution requires using a large amount of memory and extends the learning process, while not providing improvement).

The following 2D feature representations were used in this work: spectrogram, chromagram, mel spectrogram, MFCC-gram without rescaling, and MFCC-gram rescaled. All representations are resized to a resolution of 90 × 93 pixels in 4 channels (red-green-blue-amber (RGBA)). Each picture is about 0.25 s of recording, and each recording is the source of many pictures (the number of pictures depends on the content of the information about the speech signal). For example, a total of 4933 saved pictures for training results from the German recordings, i.e., 40 sentences, 8 speakers, and 2 types (in silence and noise). The number of these pictures depends on the criteria for deleting pictures without speech, pitch, and window length. All saved pictures are labeled with the gender of the speaker and whether or not there was noise during the recording of the speaker.

### 3.3. Results

#### 3.3.1. Effectiveness of 2D Feature Representations Combined with CNN for Lombard Speech Detection

According to Figure 5, several experiments were performed to show which feature representation combined with which neural network model is the most effective. The set of 2D representations used for training, validation, and testing is divided as follows: 2/3 of the whole set is used for training, out of which 7% of 2D representations are used for validation, and 1/3 of the whole set is a test set not employed in the training process. Simple network models were used to optimize training time in relation to outcomes. Topologies of the convolutional neural networks are presented in a tabular format, describing all layers and transformations. The layer annotation, along with the explanation, is shown below:

- Conv2D is a basic two-dimensional convolutional layer (a two-dimensional convolutional layer means that the input matrix is three-dimensional, representing width, height, and the number of filters).
- Max_pooling2D is a max-pooling layer, reducing the dimensions of the input layer.
- Dropout is an operation of randomly ignoring selected neurons in the learning process; this is a method of regularization and thus improves the results of learning in terms of the ability to generalize.
- Flatten is a flattening operation which means that the three-dimensional output matrix is flattened into a vector that can be used in the typical dense layer.
- Dense is a basic neural network flat layer.
- The experiments are numbered from one to nine and are presented below.

**Experiment** **1.** *Gender recognition based on the spectrogram.*

This experiment is only an initial step in recognizing Lombard speech, as it has been hypothesized that information about gender may be a vital feature supporting the detection process. The model of the CNN used is presented in Table 2. This description shows the number of filters used in every convolutional layer: the first layer contains 32 filters, and the second one 16 filters.

**Experiment** **2.** *Lombard speech detection using spectrogram*.

The same set of 2D representations was used to train the network to detect the Lombard speech type. In other words, it was a two-class classification problem. The model used to train this recognition challenge is the same as in Experiment 1 (see Table 2). The obtained accuracy in the testing set is 76%. The accuracy is not satisfying, and there are multiple wrong recognition results.

**Experiment** **3.** *Lombard speech detection using chromagram*.

In this experiment, the concept is similar to the previous one, with a different representation selected, that is chromagram. The implemented model is the same as in Experiment 1 (see Table 2). The obtained accuracy in the testing set was 58%, which is a poor result.

**Experiment** **4.** *Lombard speech detection using spectrogram with appended information about gender and with rescaling*.

In this experiment, the alpha channel was replaced with gender identification. In a “normal” image, every pixel is stored as a 4-byte information component (3 bytes for colors and 1 byte for alpha channel), and later in the training process, every byte is rescaled to the range 0 to 1. This means that because gender might be 0 or 1, the rescaled values of gender might be 0 or 1/255. It might then probably have little effect on the learning process. The CNN model is slightly changed in this experiment—the last dense layer has 512 neurons. The obtained accuracy on the testing set is 80%. The result of the experiment showed that including additional information increases classification accuracy.

**Experiment** **5.** *Lombard speech detection using spectrogram with appended gender information without rescaling*.

In this experiment, as in the previous experiment, the alpha channel was replaced with gender identification. Later, every byte value is rescaled to the 0–1 range. In this experiment, the impact of the gender bit was increased by setting its value to either 64 or 192, which means that after rescaling, its value is 0.25 or 0.75. The CNN model used is identical to Experiment 4. The obtained accuracy on the training set was 82.5%. The results of the experiment showed that the gender of the speaker might have a positive impact on the performance of the model.

**Experiment** **6.** *Lombard speech detection using chromagram with appended gender information*.

Gender information was appended similarly as in Experiment 5. The obtained accuracy on the testing set was 66%, which is inferior to other options tested.

**Experiment** **7.** *Comparison of the different types of representation and recognition performance.*

Since previous experiments showed that it is crucial to select the appropriate graphical representations and to augment the data correctly, the following approach involved testing different graphical representations and their effectiveness with comparable models and the same training time.

Various graphical representations, including a short fragment of the speech recording (approx. 0.5 s), have been tested. All 2D representations were tested with and without augmentation. Augmentation means using the alpha channel to store additional data:

- Gender of the speaker;
- F0 frequency;
- First two MFCCs.

These data are stored on a scale of 0–255 (like pixels on red-green-blue (RGB) color layers) on consecutive groups of pixels (roughly 1/4 of the transparency layer for each of the above features).

Two different models were used; they are presented in Table 3 and Table 4. Initially, a third model with an additional dense layer was tested, but it increased the general complexity of the network (increasing the number of trainable parameters) and did not improve overall accuracy.

Every model differs in several essential features: number of filters on the subsequent layers, number of neurons in the last dense layer, max-pooling size, and dropout parameter value. The network configuration used is presented in Table 5.

The convolutional neural networks prepared using the configurations presented in Table 5 were trained using the earlier discussed graphical representations. Due to space-saving, only the top fifteen scores are reported in Table 6.

The obtained results showed that the mel spectrogram with augmentation is the best candidate for further processing. The obtained accuracy is 86.72%. In contrast, the representations related to the MFCCs gave unsatisfactory results. This does not mean that the value of the features does not convey any information in this context; conversely, the augmented 2D representations use the first two MFCCs.

**Experiment** **8.** *Lombard speech detection using mel spectrogram and extended number of graphical images with augmentation.*

In the previous experiments, the number of training items was equal to the number of recordings (640) with regard to the fact that these speech excerpts were divided into training, validation, and test sets. Effectively, training was performed using 448 recordings (448 graphics), which resulted in lower recognition performance than was expected.

This time the data were prepared in the following way:

- Every speech recording was resampled using 22,050 Hz frequency;
- Average F0 was calculated for the whole file;
- Average MFCCs were calculated (second and third coefficient).

Every file was divided into windows of length around 1/3 of the sampling frequency (around 7000 samples) and the hop length of 2000 samples (which means the windows were overlapping). For each fragment, the mel spectrogram was calculated and a graphical representation was generated if at least 90% of the fragment carries energy information (to avoid training the network on segments where the majority of them contain silence). This way, 4933 mel spectrograms (each of about 7000 time-domain samples) were obtained based on 640 record files.

The model on which the network was trained (during training, the data were augmented, i.e., using a transparency layer to store information about gender, F0, and MFCCs) is presented in Table 7.

**Figure 6:**
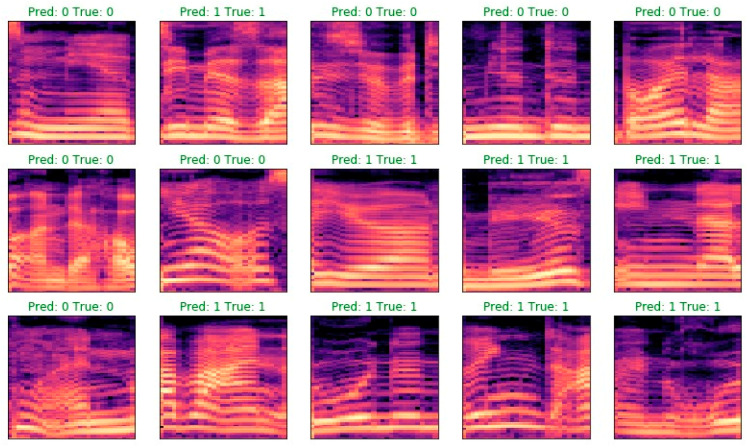
Examples of classifications for Experiment 8.

It can clearly be seen that the network has relatively few trained parameters (545,810) due to the reasonably large max pooling (3). Therefore, the number of parameters is twelve times smaller than in first model and four times smaller than in the second model. The number of epochs is 60, and the batch size is 32. Accuracy on the test set, however, is very high: 98.3%, and the loss is at 0.05. Examples of classifications are presented in Figure 6.

**Experiment** **9.** *Experiment 8 repeated without augmentation.*

**Figure 7:**
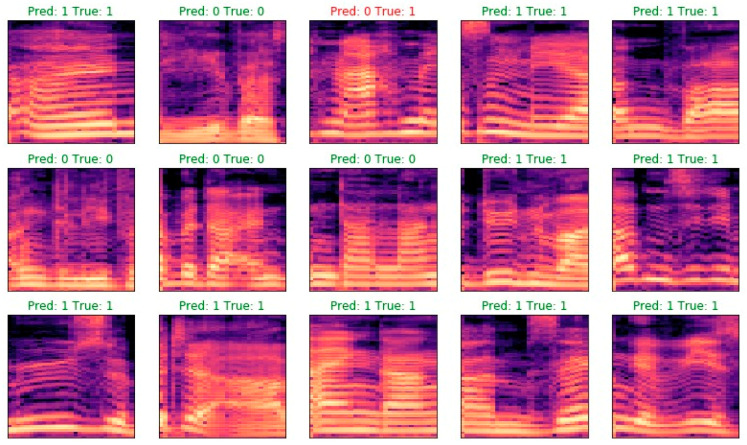
Examples of classifications for Experiment 9.

To determine to what extent augmentation is important, Experiment 8 was repeated with an identical model and hyperparameter values, but augmentation was removed from the training process. The effect is much worse—accuracy on the test set is 90% and loss is 0.23. This experiment once again confirms the importance of augmentation. Examples of classification results are presented in Figure 7.

#### 3.3.2. Evaluation of the Lombard Speech Detection Process Effectiveness

Based on the effectiveness obtained in the preliminary experiments shown in Section 3.3.1, the convolutional neural network model and the dataset presented in Experiment 8 were used for the final evaluation of the Lombard speech detection process. Therefore, implementing the detection method was divided into three stages:

***Stage 1*.** Preparation of mel spectrograms. All the images are indexed in a single file, containing—apart from the access path to the picture—information about the speaker’s gender, the presence of noise during the recording, the F0 frequency, and two MFCCs.

***Stage 2*.** Training convolutional neural network with the use of created 2D representations. Training lasts 60 epochs, and the model that provides the highest accuracy on the validation set is saved.

***Stage 3*.** Recognition tests on remaining recordings. Recognition, in this case, concerns a single fragment of the tested recording prepared in the same way as the images for training.

The entire procedure was the same for German and Polish recordings. It was assumed that processing algorithms for Lombard speech detection could not be too computationally complex, nor should they cause a longer delay in the analysis and the processing itself. For the experiments in this work, a delay of 0.5–0.7 s is acceptable.

Three convolutional neural network models, conforming to the network model structure presented in Table 7, were deployed. These models, called G1, G2, and P1, were trained and tested according to the experimental layout shown in Figure 5c. They are as follows:

- *G1 model*—a network trained on the German dataset in a proportion of 2/3 (including 7% of recordings for validation). Recordings are split into training, validation, and test sets.
- *G2 model*—a network trained on the German dataset of six speakers and tested on the other two speakers.
- *P1 model*—a network trained on the Polish dataset in a proportion of 2/3 (including 7% of recordings for validation). Recordings are split into training, validation, and test sets.

For each of them, the generation parameters are the following: the maximum frequency of the mel filter bank is 8000 Hz, and a sample divisor that affects the length of the frame used to generate it is equal to 3. 2D representation to train the neural network (the frame is 22,050 Hz/sample divider) was employed, and a shift step between frames is equal to 2000.

**Figure 8:**
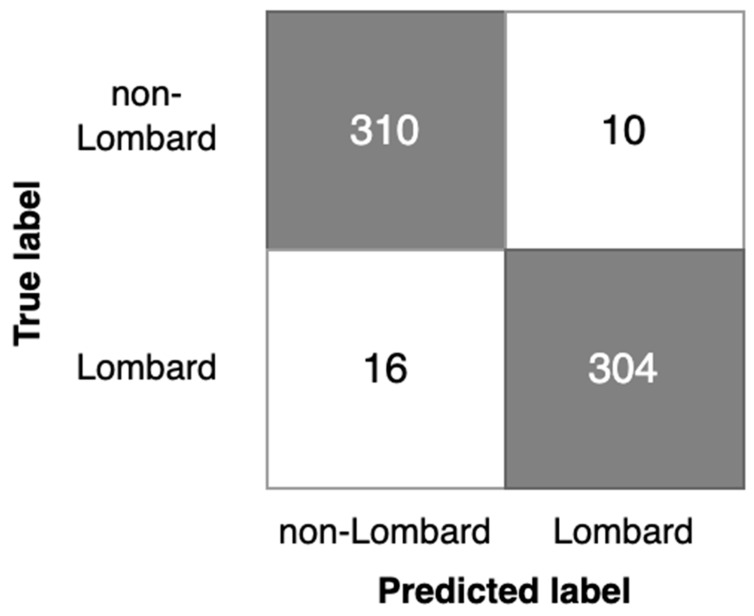
Confusion matrix for the G1 model.

**Figure 9:**
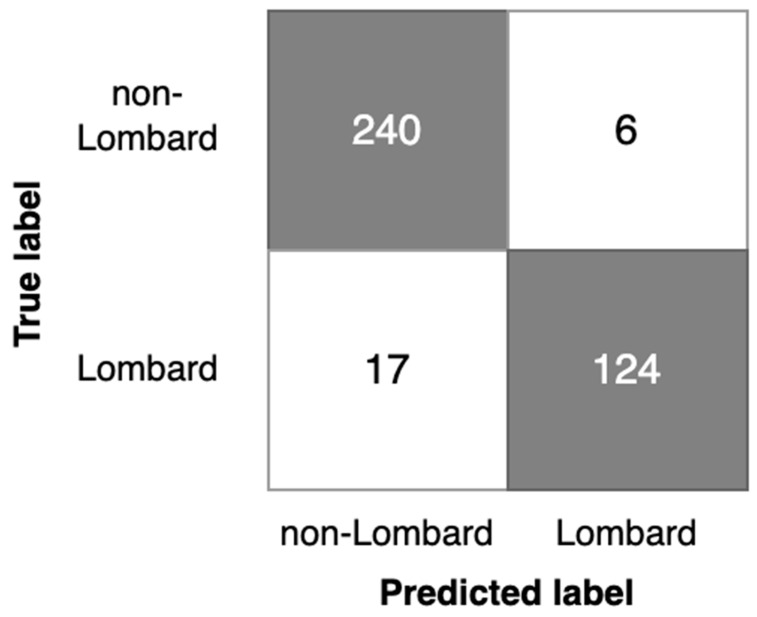
Confusion matrix for the G2 model.

**Figure 10:**
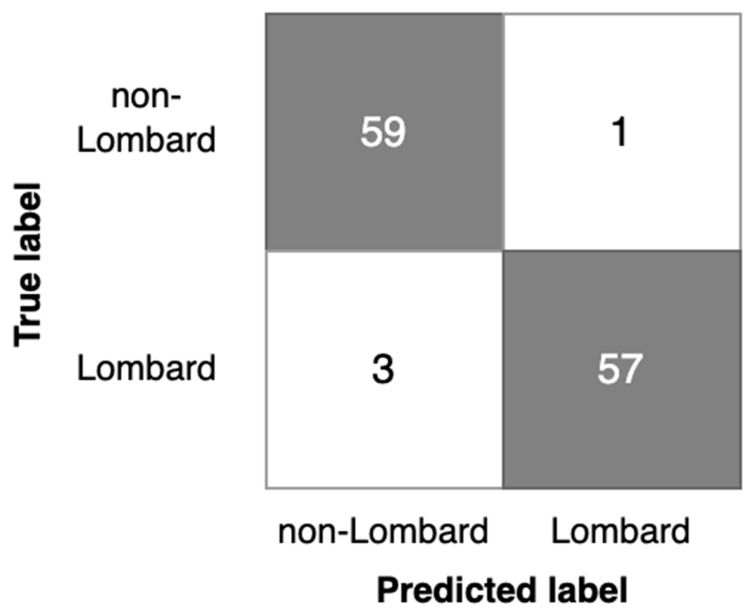
Confusion matrix for the P1 model.

The results of the Lombard speech detection based on models G1, G2, and P1 are contained in Table 8. In Figure 8, Figure 9 and Figure 10, corresponding confusion matrices are shown.

**Figure 11:**
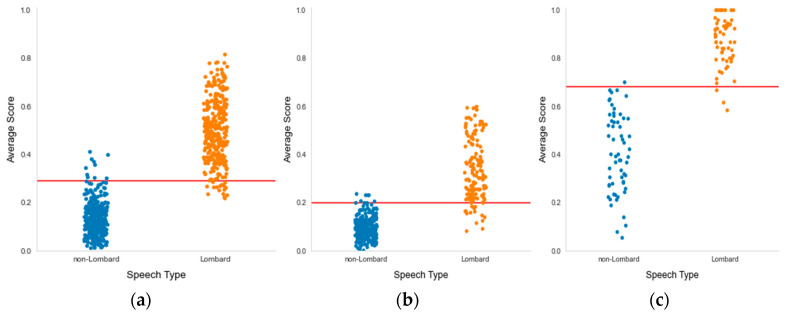
Separation plots for (**a**) the G1 model with a cutoff level of 0.29, (**b**) the G2 model with a cutoff level of 0.2, and (**c**) the P1 model with a cutoff level of 0.68.

Model value separation can be visualized using scatter plots, presenting all recognized (detected) speech types with their average detection score. Separation plots are presented in Figure 11.

As seen from the above charts (Figure 8), there is a clear separation between Lombard and neutral recordings with the threshold applied. It can successfully be used to implement the near real-time decision system component.

#### 3.3.3. Discussion

The Lombard effect’s impact on speech signal analysis-based technologies is well-known and explored [13,14,15,16,17,18,19,20]. The variability of resulting speech-in-noise characteristics causes lower speech intelligibility and reduces accuracy in speech recognition, speaker identification and verification, speech emotion recognition, and other speech signal-related tasks [40]. Increasing speech signal variability is typically compensated by larger training datasets, additional adaptation, and variability modeling. In machine learning-based processing, this is carried out by incrementing training data. A standard solution is to artificially increase the quantity of training data patterns by transforming the available speech patterns by adding noise, time warping and shifting, pitch shifting, time or frequency masking, or filtering [58,59,60].

In this study, we proposed and explored another approach using additional features related to the speaker’s acoustic characteristics to augment the training data. Features such as gender, which determines the pitch of the speech signal, the fundamental frequency, its variability, and other speaker-dependent characteristics, can help to characterize the personal acoustic properties separately from changes caused by the Lombard effect. Successful identification and incorporation of these features mean we can augment training data with additional parameters, thereby increasing the ability to identify the Lombard effect. To achieve this goal, we have conducted a series of experiments.

The starting question concerns the 2D representation of the speech signal and its acoustic properties, i.e., which of the available signal analysis techniques can help to determine the highest accuracy in detecting Lombard speech? In our investigation, we have evaluated spectrograms, mel spectrograms, chromagrams, and MFCC-grams as potential techniques for the 2D representation of speech signals. Our experiments indicate the superiority of mel scale and linear frequency spectrograms (see Table 6). In the case of the mel spectrogram, the highest Lombard effect detection accuracy of 86.7% was obtained, with the 6 following best results achieved when employing spectrograms (81.3–85.2%). The result is not surprising, as the raw spectrogram data fully describe the speech signal spectral properties over time. Furthermore, in some cases, additionally processed spectrograms such as mel-scaled or bandpass-filtered spectrograms can gain superiority due to increased robustness and noise removal, as seen in the results in Table 6.

The next point discussed relates to which acoustic (or any other) feature can be selected for data augmentation. Previous studies demonstrated that environmental noise affects speakers differently [40]. The interspeaker variability in the magnitude of Lombard response is shown in [61], where recordings of five male and five female subjects were analyzed. N. Alghamdi and her colleagues [62] showed that gender differences were also noticed in the extent of the Lombard effect. For example, female talkers have shown a greater increase in loudness, estimated vowel duration, estimated vowel-to-utterance ratio, and mouth aperture, as well as a more considerable decrease in vowel formant F2 frequency. The differences between genders when it comes to Lombard speech were analyzed in the work of Kleczkowski et al. [63]. The separate analysis of males’ and females’ speech revealed that the latter increased their vocal effort more (by 8.07 dB on average) than males (6.65 dB). Although the gender differences in Lombard speech are widely described in the literature, such information is not implemented in real-time systems.

Considering these findings, we have selected the following features: binary parameter related to the speaker gender, averaged fundamental frequency F0 value, and the averaged first two MFCC coefficients, which are associated with the content of the speech utterance. We believe these are the features reflecting the speaker and the acoustic characteristics of the speaker’s speech. To verify the adequacy of the augmented representation, 4933 augmented mel spectrograms were extracted from 640 recorded utterances on which the CNN-based model was trained. Under these conditions, the Lombard speech detection accuracy reached 98.3% (Experiment 8). Additionally, the model was tested on German and Polish speech utterances. Under various testing conditions, the Lombard effect recognition rates were 95.9% and 96.7%, respectively. In all cases, the obtained recognition accuracy was significantly higher than the rate of 90% achieved using non-augmented spectrograms (Experiment 9). Thus, using acoustic characteristics of the speakers facilitates the recognition of modified speech. Furthermore, these features also provide knowledge about language characteristics, as both German and Polish cases show similar accuracy rates. However, the complete versatility and robustness of augmented mel spectrograms should be evaluated additionally with a wider variety of languages.

Considering the results obtained, the following generalized key points are derived from Lombard speech detection investigation:

- Analysis of detection errors (Figure 8, Figure 9 and Figure 10) shows the predominance of false negative errors (i.e., Lombard speech was identified as non-Lombard). The error rate was 1.6–3 times higher than the rate of false positive type errors (non-Lombard speech was detected as Lombard). This may be due to the highly specific characteristics of some speakers or the insufficient discriminant power of augmented features. In the latter case, the study of 2D representation augmentation should be continued in the search for additional features.
- The investigated setup enabled the near real-time detection of the Lombard effect. An operational delay of 0.5–0.7 s was found during the investigation, which is acceptable for real-world applications.
- Gender information should be used to identify the Lombard speech. Therefore, it is necessary to consider an automated gender identification stage, preferably using a separate classification model. Our experimental results show 93% accuracy of spectrogram-based speaker gender identification (Experiment 1), which may be sufficient for Lombard speech identification.
- The detection process should also consider the silence between utterances and be capable of disregarding these fragments. Possible solutions for deployment are as follows: the Lombard speech identification process may be extended to a three-valued classification, i.e., non-Lombard speech, Lombard speech, or a voice activity detection (VAD) algorithm should be used separately as a self-sufficient component supporting silence detection.
- The implemented Lombard speech detection setup requires defining the cutoff value between Lombard and non-Lombard speech. These values differ for different datasets (Figure 11). The automatic definition of this value is one of the challenges for future research.

## 4. Conclusions

In this paper, it was shown that deep learning based on convolutional neural networks (CNN) is capable of detecting Lombard speech effectively. Interestingly, CNN provides convincing results even when the differences between Lombard and neutral (non-Lombard) speech detected by the network are difficult to discern with an “expert eye”.

The obtained classification accuracy on the test set was 98.3%.

Overall, the evaluation of the results of the automatic Lombard speech detection process revealed a clear separation between Lombard and neutral recordings. The obtained deep model accuracies are the following: 95.94%, 94.06%, and 96.67%, depending on the dataset employed.

Moreover, mel spectrograms were used as images in the recognition process. These images are not generated as physical files but as in-memory visualizations; generated visualizations are stored in the memory as a byte array. However, from the point of view of the convolutional network, creating physical files is not necessary as CNN treats data in the same way, whether it is an image or an ordinary data tensor. Therefore, resigning to visualization will accelerate the recognition process, so this is one of the future research directions.

In the experiments, Lombard speech detection was performed for the entire recording. If such a component is to be used in real-time systems, the recognition process should also be performed in real time, however, with a short delay at the start. This means that before the recognition decision is made, there must be a short time interval to collect a part of the speech signal to perform detection. This leads to another possible improvement in developing a robust averaging algorithm for practical applications.

The last remark concerns the challenge of insufficient resources to train more complex deep models; thus, the effort should be on creating synthetic Lombard speech as it happens now in ASR systems [64].

Therefore, future work can take advantage of the above-indicated limitations to make the Lombard speech detection process more robust in future investigations.

## Figures and Tables

**Table 1:** Sets of recordings used for training and inference.

Language	Set Details
German (Soloducha et al., 2016) [56]	40 s-long statements 8 speakers (including 3 females and 5 males) Each sentence was recorded under conditions of silence and with accompanying disturbance
Polish (Czyzewski et al., 2017) [57]	15 s-long statements 4 speakers (including 2 females and 2 males) Each sentence was recorded under conditions of silence and with accompanying disturbance.

**Table 2:** Model of the network used in Experiment 1.

Layer (Type)	Output Shape	Number of Parameters
conv2d_2 (Conv2D)	(None, 90, 93, 32)	544
max_pooling2d_2 (MaxPooling2D)	(None, 45, 46, 32)	0
dropout_3 (Dropout)	(None, 45, 46, 32)	0
conv2d_3 (Conv2D)	(None, 45, 46, 16)	2064
max_pooling2d_3 (MaxPooling2D)	(None, 22, 23, 16)	0
dropout_4 (Dropout)	(None, 22, 23, 16)	0
flatten_1 (Flatten)	(None, 8096)	0
dense_2 (Dense)	(None, 256)	2,072,832
dropout_5 (Dropout)	(None, 256)	0
dense_3 (Dense)	(None, 2)	514

The obtained accuracy in the testing set is 93%.

**Table 3:** The first model of the network used in Experiment 7.

Layer (Type)	Output Shape	Number of Parameters
conv2d_12 (Conv2D)	(None, 90, 93, 32)	2080
max_pooling2d_12 (MaxPooling2D)	(None, 45, 46, 32)	0
dropout_20 (Dropout)	(None, 45, 46, 32)	0
conv2d_13 (Conv2D)	(None, 45, 46, 48)	24,624
max_pooling2d_13 (MaxPooling2D)	(None, 22, 23, 48)	0
dropout_21 (Dropout)	(None, 22, 23, 48)	0
flatten_4 (Flatten)	(None, 24,288)	0
dense_12 (Dense)	(None, 256)	6,217,984
dropout_22 (Dropout)	(None, 256)	0
dense_13 (Dense)	(None, 2)	514

**Table 4:** The second model of the network used in Experiment 7.

Layer (Type)	Output Shape	Number of Parameters
conv2d_14 (Conv2D)	(None, 90, 93, 32)	1184
max_pooling2d_14 (MaxPooling2D)	(None, 45, 46, 32)	0
dropout_23 (Dropout)	(None, 45, 46, 32)	0
conv2d_15 (Conv2D)	(None, 45, 46, 48)	13,872
max_pooling2d_15 (MaxPooling2D)	(None, 22, 23, 48)	0
dropout_24 (Dropout)	(None, 22, 23, 48)	0
conv2d_16 (Conv2D)	(None, 22, 23, 64)	27,712
max_pooling2d_16 (MaxPooling2D)	(None, 11, 11, 64)	0
dropout_25 (Dropout)	(None, 11, 11, 64)	0
flatten_5 (Flatten)	(None, 7744)	0
dense_14 (Dense)	(None, 256)	1,982,720
dropout_26 (Dropout)	(None, 256)	0
dense_15 (Dense)	(None, 2)	514

**Table 5:** Configuration of the learning process for speech type detection.

ID	Model According to Table 3 (1st Model) and Table 4 (2nd Model)	Number of Filters in Conv. Layers	Size of Kernel	Max Pooling	Dropout after Conv. Layers	Dropout after Dense Layer	Number of Neurons in the Dense Layer	Batch Size	Number of Epochs
1	1	16, 32	2	2	0.3	0.5	256	64	35
2	1	32, 48	2	2	0.3	0.5	256	64	35
3	1	32, 48	3	2	0.3	0.5	256	64	35
4	1	32, 48	3	2	0.3	0.5	256	32	35
5	1	32, 48	5	2	0.3	0.5	256	64	35
6	1	32, 48	5	2	0.3	0.5	256	32	35
7	2	32, 48, 64	3	2	0.3	0.5	256	64	35
8	2	32, 48, 64	5	3	0.3	0.5	256	64	35
9	2	32, 48, 64	5	3	0.3	0.5	256	32	35

**Table 6:** A summary of the results of the speech type detection experiments.

Result No.	Type of Graphical Representation Employed	Augmented/Clean Picture	Experiment Configuration According to ID from Table 5	Accuracy of the Testing Set
1	Mel spectrogram	Augmented	9	0.8671875
2	Spectrogram	Clean	8	0.8515625
3	Spectrogram	Clean	6	0.84375
4	Spectrogram	Clean	4	0.8359375
5	Spectrogram	Augmented	8	0.828125
6	Spectrogram	Augmented	9	0.828125
7	Spectrogram	Augmented	7	0.8125
8	Mel spectrogram	Clean	6	0.8125
9	Spectrogram	Clean	7	0.8046875
10	Spectrogram	Augmented	5	0.8046875
11	Mel spectrogram	Clean	7	0.8046875
12	Mel spectrogram	Augmented	6	0.8046875
13	Mel spectrogram	Clean	5	0.796875
14	Spectrogram	Clean	5	0.7890625
15	Mel spectrogram	Clean	8	0.7890625

**Table 7:** Model of the network used in Experiment 8.

Layer (Type)	Output Shape	Number of Parameters
conv2d_20 (Conv2D)	(None, 90, 93, 32)	3232
max_pooling2d_20 (MaxPooling2D)	(None, 30, 31, 32)	0
dropout_32 (Dropout)	(None, 30, 31, 32)	0
conv2d_21 (Conv2D)	(None, 30, 31, 48)	38,448
max_pooling2d_21 (MaxPooling2D)	(None, 10, 10, 48)	0
dropout_33 (Dropout)	(None, 10, 10, 48)	0
conv2d_22 (Conv2D)	(None, 10, 10, 64)	76,864
max_pooling2d_22 (MaxPooling2D)	(None, 3, 3, 64)	0
dropout_34 (Dropout)	(None, 3, 3, 64)	0
flatten_7 (Flatten)	(None, 576)	0
dense_19 (Dense)	(None, 512)	295,424
dropout_35 (Dropout)	(None, 512)	0
dense_20 (Dense)	(None, 256)	131,328
dropout_36 (Dropout)	(None, 256)	0
dense_21 (Dense)	(None, 2)	514

**Table 8:** Lombard speech detection results.

Model	G1	G2	P1
Number of samples used for training	3156	2334	816
Number of samples used for validation	790	584	205
Accuracy of the validation set	0.9899	0.9880	0.9902
Loss of the validation set	0.0370	0.0434	0.0432
Cutoff level	0.29	0.20	0.68
Recognition accuracy	0.9594	0.9406	0.9667
Precision	0.9681	0.9538	0.9828
Recall	0.9500	0.8794	0.9500

## Data Availability

Not applicable.

## References

[1] Lombard E. (1911). Le signe de l’elevation de la voix. Ann. Mal. De L’Oreille Et Du Larynx.

[2] Junqua J.-C. (1996). The influence of acoustics on speech production: A noise-induced stress phenomenon known as the Lombard reflex. Speech Commun..

[3] Amazi D.K., Garber S.R. (1982). The Lombard sign as a function of age and task. J. Speech Lang. Hear. Res..

[4] Khan M.N., Naseer F. (2020). IoT based university garbage monitoring system for healthy environment for students. Proceedings of the 14th International Conference on Semantic Computing (ICSC).

[5] Jamil M., Khan M.N., Rind S.J., Awais Q., Uzair M. (2021). Neural network predictive control of vibrations in tall structure: An experimental controlled vision. Comput. Electr. Eng..

[6] Justus D., Brennan J., Bonner S., McGough A.S. Predicting the computational cost of deep learning models. Proceedings of the 2018 IEEE International Conference on Big Data (Big Data).

[7] Lopez-Ballester J., Pastor-Aparicio A., Felici-Castell S., Segura-Garcia J., Cobos M. (2020). Enabling real-time computation of psycho-acoustic parameters in acoustic sensors using convolutional neural networks. IEEE Sens. J..

[8] He Y., Dong X. (2020). Real time speech recognition algorithm on embedded system based on continuous Markov model. Microprocess. Microsyst..

[9] Phruksahiran N. (2022). Audio Feature and Correlation Function-Based Speech Recognition in FM Radio Broadcasting. ECTI Transactions on Electrical Engineering, Electron. Commun..

[10] Bottalico P., Piper R.N., Legner B. (2022). Lombard effect, intelligibility, ambient noise, and willingness to spend time and money in a restaurant amongst older adults. Sci. Rep..

[11] Hansen J.H., Lee J., Ali H., Saba J.N. (2020). A speech perturbation strategy based on “Lombard effect” for enhanced intelligibility for cochlear implant listeners. J. Acoust. Soc. Am..

[12] Ngo T., Kubo R., Akagi M. (2021). Increasing speech intelligibility and naturalness in noise based on concepts of modulation spectrum and modulation transfer function. Speech Commun..

[13] Boril H., Hansen J.H. (2009). Unsupervised equalization of Lombard effect for speech recognition in noisy adverse environments. IEEE Trans. Audio Speech Lang. Process..

[14] Heracleous P., Ishi C.T., Sato M., Ishiguro H., Hagita N. (2013). Analysis of the visual Lombard effect and automatic recognition experiments. Comput. Speech Lang..

[15] Marxer R., Barker J., Alghamdi N., Maddock S. (2018). The impact of the Lombard effect on audio and visual speech recognition systems. Speech Commun..

[16] Vlaj D., Kacic Z. (2011). The influence of Lombard effect on speech recognition. Speech Technologies.

[17] Kelly F., Hansen J.H. Evaluation and calibration of Lombard effects in speaker verification. Proceedings of the 2016 IEEE Spoken Language Technology Workshop (SLT).

[18] Kelly F., Hansen J.H. (2021). Analysis and calibration of Lombard effect and whisper for speaker recognition. IEEE/ACM Trans. Audio Speech Lang. Process..

[19] Saleem M.M., Liu G., Hansen J.H. Weighted training for speech under Lombard effect for speaker recognition. Proceedings of the 40th IEEE International Conference on Acoustics, Speech and Signal Processing (ICASSP).

[20] Zhao Y., Ando A., Takaki S., Yamagishi J., Kobashikawa S. Does the Lombard Effect Improve Emotional Communication in Noise?-Analysis of Emotional Speech Acted in Noise. In Proceedings of the INTERSPEECH.

[21] Junqua J.-C. (1993). The Lombard reflex and its role on human listeners and automatic speech recognizers. J. Acoust. Soc. Am..

[22] Kisic D., Horvat M., Jambrošic K., Francek P. (2022). The Potential of Speech as the Calibration Sound for Level Calibration of Non-Laboratory Listening Test Setups. Appl. Sci..

[23] Ma P., Petridis S., Pantic M. Investigating the Lombard effect influence on end-to-end audio-visual speech recognition. Proceedings of the Interspeech.

[24] Steeneken H.J.M., Hansen J.H.L. Speech under stress conditions: Overview of the effect on the speech production and on system performance. Proceedings of the IEEE International Conference on Acoustics, Speech, and Signal Processing Proceedings (ICASSP99).

[25] Kurowski A., Kotus J., Odya P., Kostek B. (2022). A Novel Method for Intelligibility Assessment of Nonlinearly Processed Speech in Spaces Characterized by Long Reverberation Times. Sensors.

[26] Cooke M., Lecumberri M.L.G. (2012). The intelligibility of Lombard speech for non-native listeners. J. Acoust. Soc. Am..

[27] Marcoux K., Cooke M., Tucker B.V., Ernestus M. (2022). The Lombard intelligibility benefit of native and non-native speech for native and non-native listeners. Speech Commun..

[28] Summers W.V., Pisoni D.B., Bernacki R.H., Pedlow R.I., Stokes M.A. (1988). Effects of noise on speech production: Acoustic and perceptual analyses. J. Acoust. Soc. Am..

[29] Trabelsi A., Warichet S., Aajaoun Y., Soussilane S. (2022). Evaluation of the efficiency of state-of-the-art Speech Recognition engines. Procedia Comput. Sci..

[30] Abdusalomov A.B., Safarov F., Rakhimov M., Turaev B., Whangbo T.K. (2022). Improved Feature Parameter Extraction from Speech Signals Using Machine Learning Algorithm. Sensors.

[31] Ogundokun R.O., Abikoye O.C., Adegun A.A., Awotunde J.B. (2020). Speech Recognition System: Overview of the State-Of-The-Arts. Int. J. Eng. Res. Technol..

[32] Hannun A., Case C., Casper J., Catanzaro B., Diamos G., Elsen E., Prenger R., Satheesh S., Sengupta S., Coates A. (2014). Deep speech: Scaling up end-to-end speech recognition. arXiv.

[33] Povey D., Ghoshal A., Boulianne G., Burget L., Glembek O., Goel N., Hannemann M., Motlicek P., Qian Y., Schwarz P. The kaldi speech recognition toolkit. Proceedings of the IEEE 2011 Workshop on Automatic Speech Recognition and Understanding.

[34] Che G., Chai S., Wang G.-B., Du J., Zhang W.-Q., Weng C., Su D., Povey D., Trmal J., Zhang J. GigaSpeech: An Evolving, Multi-Domain ASR Corpus with 10,000 Hours of Transcribed Audio. Proceedings of the INTERSPEECH 2021.

[35] Ezzerg A., Gabrys A., Putrycz B., Korzekwa D., Trigueros D.S., McHardy D., Pokora K., Lachowicz J., Trueba J.L., Klimkov V. (2021). Enhancing Audio Quality for Expressive Neural Text-to-Speech. arXiv.

[36] Jiao Y., Gabryś A., Tinchev G., Putrycz B., Korzekwa D., Klimkov V. Universal neural vocoding with parallel wavenet. Proceedings of the ICASSP 2021 IEEE International Conference on Acoustics, Speech and Signal Processing (ICASSP).

[37] Merritt T., Ezzerg A., Biliński P., Proszewska M., Pokora K., Barra-Chicote R., Korzekwa D. (2022). Text-Free Non-Parallel Many-to-Many Voice Conversion Using Normalising Flows. arXiv.

[38] Nossier S.A., Wall J., Moniri M., Glackin C., Cannings N. (2021). An Experimental Analysis of Deep Learning Architectures for Supervised Speech Enhancement. Electronics.

[39] Korvel G., Kąkol K., Kurasova O., Kostek B. (2020). Evaluation of Lombard speech models in the context of speech in noise enhancement. IEEE Access.

[40] Zhang J., Zorila C., Doddipatla R., Barker J. (2022). On Monoaural Speech Enhancement for Automatic Recognition of Real Noisy Speech Using Mixture Invariant Training. arXiv.

[41] Furoh T., Fukumori T., Nakayama M., Nishiura T. (2013). Detection for Lombard speech with second-order mel-frequency cepstral coefficient and spectral envelope in beginning of talking-speech. Proceedings of the Meetings on Acoustics ICA2013).

[42] Goyal J., Khandnor P., Aseri T.C. (2020). Classification, prediction, and monitoring of Parkinson’s disease using computer assisted technologies: A comparative analysis. Eng. Appl. Artif. Intell..

[43] Scharf M.K., Hochmuth S., Wong L.L., Kollmeier B., Warzybok A. (2022). Lombard Effect for Bilingual Speakers in Cantonese and English: Importance of Spectro-Temporal Features. arXiv.

[44] Piotrowska M., Korvel G., Kostek B., Ciszewski T., Czyzewski A. (2019). Machine learning-based analysis of English lateral allophones. Int. J. Appl. Math. Comput. Sci..

[45] Piotrowska M., Czyzewski A., Ciszewski T., Korvel G., Kurowski A., Kostek B. (2021). Evaluation of aspiration problems in L2 English pronunciation employing machine learning. J. Acoust. Soc. Am..

[46] Korvel G., Treigys P., Tamulevicius G., Bernataviciene J., Kostek B. (2018). Analysis of 2D Feature Spaces for Deep Learning-Based Speech Recognition. J. Audio Eng. Soc..

[47] Vafeiadis A., Votis K., Giakoumis D., Tzovaras D., Chen L., Hamzaoui R. (2020). Audio content analysis for unobtrusive event detection in smart homes. Eng. Appl. Artif. Intell..

[48] Tamulevičius G., Korvel G., Yayak A.B., Treigys P., Bernatavičienė J., Kostek B. (2020). A study of cross-linguistic speech emotion recognition based on 2D feature spaces. Electronics.

[49] Tariq Z., Shah S.K., Lee Y. Speech Emotion Detection using IoT based Deep Learning for Health Care. Proceedings of the IEEE International Conference on Big Data (Big Data).

[50] Er M.B., Isik E., Isik I. (2021). Parkinson’s detection based on combined CNN and LSTM using enhanced speech signals with variational mode decomposition. Biomed. Signal Process. Control..

[51] Almeida J.S., Rebouças Filho P.P., Carneiro T., Wei W., Damaševičius R., Maskeliūnas R., de Albuquerque V.H.C. (2019). Detecting Parkinson’s disease with sustained phonation and speech signals using machine learning techniques. Pattern Recognit. Lett..

[52] Laguarta J., Hueto F., Subirana B. (2020). COVID-19 artificial intelligence diagnosis using only cough recordings. IEEE Open J. Eng. Med. Biol..

[53] Gu J., Wang Z., Kuen J., Ma L., Shahroudy A., Shuai B., Liu T., Wang X., Wang G., Cai J. (2018). Recent advances in convolutional neural networks. Pattern Recognit..

[54] LeCun Y., Haffner P., Bottou L., Bengio Y. (1999). Object Recognition with Gradient-Based Learning. Shape, Contour and Grouping in Computer Vision.

[55] Müller M. (2015). Fundamentals of Music Processing: Audio, Analysis, Algorithms, Applications.

[56] Soloducha M., Raake A., Kettler F., Voigt P. Lombard speech database for German language. Proceedings of the 42nd Annual Conference on Acoustics (DAGA).

[57] Czyzewski A., Kostek B., Bratoszewski P., Kotus J., Szykulski M. (2017). An audio-visual corpus for multimodal automatic speech recognition. J. Intell. Inf. Syst..

[58] Park D.S., Chan W., Zhang Y., Chiu C.-C., Zoph B., Cubuk E.D., Le Q.V. SpecAugment: A Simple Data Augmentation Method for Automatic Speech Recognition. Proceedings of the Interspeech 2019.

[59] Song X., Wu Z., Huang Y., Su D., Meng H. SpecSwap: A Simple Data Augmentation Method for End-to-End Speech Recognition. Proceedings of the Interspeech 2020.

[60] Abayomi-Alli O.O., Damaševičius R., Qazi A., Adedoyin-Olowe M., Misra S. (2022). Data Augmentation and Deep Learning Methods in Sound Classification: A Systematic Review. Electronics.

[61] Junqua J.C., Fincke S., Field K. The Lombard effect: A reflex to better communicate with others in noise. Proceedings of the 1999 IEEE International Conference on Acoustics, Speech, and Signal Processing, ICASSP99 (Cat. No. 99CH36258).

[62] Alghamdi N., Maddock S., Marxer R., Barker J., Brown G.J. (2018). A corpus of audio-visual Lombard speech with frontal and profile views. J. Acoust. Soc. Am..

[63] Kleczkowski P., Żak A., Król-Nowak A. (2017). Lombard effect in Polish speech and its comparison in English speech. Arch. Acoust..

[64] Korzekwa D., Lorenzo-Trueba J., Drugman T., Kostek B. (2022). Computer-assisted pronunciation training—Speech synthesis is almost all you need. Speech Commun..

